# The use of enoxaparin as bridge to therapeutic INR after LVAD implantation

**DOI:** 10.1186/s13019-020-01373-y

**Published:** 2020-11-14

**Authors:** Zubair Shah, Ioannis Mastoris, Prakash Acharya, Aniket S. Rali, Moghni Mohammed, Sami Farhad, Sagar Ranka, Savahanna Wagner, Giorgio Zanotti, Christopher T. Salerno, Nicholas A. Haglund, Andrew J. Sauer, Ashwin K. Ravichandran, Travis Abicht

**Affiliations:** 1 University of Kansas Health System, University of Kansas School of Medicine, Department of Cardiovascular Medicine, Kansas City, Kansas USA; 2 Cardiovascular Service Line, Ascension, St. Vincent Hospital, Indianapolis, Indiana USA; 3 University of Kansas Health System, University of Kansas School of Medicine, Department of Cardiovascular and Thoracic Surgery, Kansas City, Kansas USA

**Keywords:** Low molecular weight heparin, Unfractionated heparin, Left ventricular assist device, Bridging anticoagulation

## Abstract

**Background:**

Left ventricular assist devices (LVAD) have been increasingly used in the treatment of end-stage heart failure. While warfarin has been uniformly recommended in the long-term as anticoagulation strategy, no clear recommendation exists for the post-operative period. We sought to evaluate the feasibility of enoxaparin in the immediate and early postoperative period after LVAD implantation.

**Methods:**

This is a two-center, retrospective analysis of 250 consecutive patients undergoing LVAD implantation between January 2017 and December 2018. Patients were bridged postoperatively to therapeutic INR by either receiving unfractionated heparin (UFH) or low molecular weight heparin (LMWH). Patients were followed while inpatient and for 3 months after LVAD implantation. The efficacy outcome was occurrence of first and subsequent cerebrovascular accident while safety outcome was the occurrence of bleeding events. Length of stay (LOS) was also assessed.

**Results:**

Two hundred fifty and 246 patients were analyzed for index admission and 3-month follow up respectively. No statistically significant differences were found between the two groups in CVA (OR = 0.67; CI = 0.07–6.39, *P* = 0.73) or bleeding events (OR = 0.91; CI = 0.27–3.04, *P* = 0.88) during index admission. Similarly, there were no differences at 3 months in either CVAs or bleeding events (OR = 0.85; 0.31–2.34; *p* = 0.76). No fatal events occurred during the study follow-up period. Median LOS was significantly lower (4 days; *p* = 0.03) in the LMWH group.

**Conclusions:**

LMWH in the immediate and early postoperative period after LVAD implantation appears to be a concurrently safe and efficacious option allowing earlier postoperative discharge and avoidance of recurrent hospitalizations due to sub-therapeutic INR.

## Introduction

Implantation of left ventricular assist devices (LVADs) in treatment of end-stage heart failure has consistently been increasing over the past decade with current annual implant rates exceeding 2500/year [1]. Although LVADs have revolutionized the treatment of end-stage heart failure, serving either as destination therapy or as a bridge to transplantation, various clinical challenges and complications are associated with their use. These include pump thrombosis, increased rates of bleeding events associated with anticoagulation use, and cerebral vascular accidents (CVAs) [2, 3]. To prevent thromboembolic events associated with pump function, early initiation of therapeutic anticoagulation is recommended in the post-operative period (< 24 h) [4–6]. Vitamin K antagonists are the mainstay of long-term anti-coagulation therapy in LVAD patients. In contrast, the parenteral anticoagulation regimen following LVAD implantation has been less well-defined and studied. The current 2013 International Society of Heart and Lung Transplantation consensus guidelines for mechanical circulatory support recommend Warfarin as the first line anti-coagulant for long-term anticoagulation and intravenous (IV) unfractionated heparin (UFH) or other IV anticoagulation therapy as a bridging method [7]. In contrast the 2019 EACTS Expert Consensus on long-term mechanical circulatory support solely recommend treatment with IV heparin as bridge to therapeutic INR in addition to background antiplatelet therapy [8, 9]. These guidelines have been aligned with the recommended anticoagulation regimen of IV heparin used post-operatively as bridge to device-specific INR in the HeartMate II Pivotal Trial and the ADVANCE and ENDURANCE Heartware trials; however, no specific recommendations were written into the MOMENTUM 3 trial except in the case of pump thrombosis [10, 11].

Low molecular weight heparins (LMWH) such as enoxaparin or fondaparinux have emerged as an attractive alternative to UFH in various clinical settings including prevention and treatment of pulmonary embolism, treatment of acute coronary syndromes or as a bridging method in patients with planned surgical procedures and indication for anticoagulation [12–14]. LMWH provides a more predictable anticoagulation pattern, does not necessarily require regular monitoring and can be easily used in the outpatient setting for anticoagulation purposes, consequentially reducing length of stay (LOS) and re-admissions for bridging. The use of LMWH after major cardiac surgery for valve replacement has rendered some favorable outcomes, but the role of LMWH in the setting of LVAD has not been elucidated [15–17].

There is a paucity of data on the safety and efficacy of LMWH as bridge to therapeutic device-specific INR in the immediate and early post-surgical period. The aim of our study is to evaluate the safety and efficacy of enoxaparin in the immediate period after LVAD implantation and up to 3 months post-operatively as a bridging strategy to therapeutic INR in consecutive patients undergoing LVAD implantation.

## Methods

### Study population

This is a two-center retrospective analysis of 250 consecutive patients with end-stage heart failure undergoing either HVAD (HeartWare International Inc., Framingham, MA), HeartMate II or HeartMate III (Abbott Corporation; Plymouth, MN) LVAD implantation between January 2017 and December 2018 at The University of Kansas Health System in Kansas City, Kansas and at Ascension St. Vincent in Indianapolis, Indiana. Patients were stratified according to the anticoagulation strategy used in enoxaparin (Lovenox; Sanofi) or UFH groups and timing of bridging (index hospitalization [immediate postoperative] vs within 3-months follow-up [early postoperative]). Patients were bridged with LMWH either on index admission, during the 3 month follow up period (should that have been required) or both. Patients with suspected or confirmed heparin induced thrombocytopenia were excluded from the study. Patients were followed daily in the inpatient setting and in planned clinic visits per each institution protocol post LVAD implantation. Data were collected in a retrospective manner from KUMC and St. Vincent prospective LVAD registries and supplemented by chart review of patients’ electronic medical records.

### Anti-coagulation protocol

All patients were initiated on anticoagulation with either LMWH or UFH within 24 h and preferably within 12 h post LVAD implantation. Anticoagulation initiation was delayed if there was concern for post-operative bleeding. Most patients post-implantation are initiated on UFH in case of early bleeding complications. The initial dose of LMWH was 0.5 mg/kg administered subcutaneously every 12 h. Unfractionated heparin was initiated as a continuous intravenous infusion using standard protocol with initial bolus at the discretion of the physician. Peak anti-Xa activity of 0.2 to 0.4 IU/ml and PTT level of 60–80 s were considered therapeutic. LMWH dose was adjusted accordingly when anti-Xa levels were not within the target range. Similarly, UFH infusion rate was titrated for a goal PTT level of 60–80 s, or as clinically tolerated. LMWH or UFH was continued until a target international normalized ratio (INR) of 2 to 2.5 was achieved with Warfarin. Warfarin was started when deemed appropriate from a surgical perspective which was generally in the evening of postoperative day 1. Anti-platelet therapy with Aspirin 81–325 mg/day was initiated on the day of implant unless deemed surgically unsafe due to bleeding concerns. Patients that have been deemed ready for discharge but had not yet achieved a therapeutic INR were either discharged on enoxaparin or transitioned to enoxaparin from UFH for discharge. Patients were followed daily while inpatient and per hospital protocol after discharge in an established VAD clinic for the first 3 months post discharge from index hospitalization. Those that were found to have a sub-therapeutic INR (after having achieved a therapeutic INR post operatively) during routine follow-up would either be either be prescribed therapeutic enoxaparin or admitted to the hospital for IV UFH therapy per physician discretion. Patients that were elected to be treated as outpatient had increased INR monitoring until therapeutic levels were achieved.

### Study outcomes

The primary efficacy outcome was defined as the occurrence of any cerebrovascular accident (ischemic and hemorrhagic) in the first 3 months post LVAD implantation. The primary safety outcome was defined as the occurrence of any clinically significant bleeding event prompting at least hospitalization for inpatient monitoring. Bleeding events that occurred in the immediate post-operative period from the first day of parenteral anticoagulation, deemed non-surgical in nature were also included in the analysis. Those included thoracic, retroperitoneal, gastrointestinal and any bleeding of unknown source. Bleeding for the purposes of this study was defined as any overt bleeding or acute drop in hemoglobin without an identifiable source requiring a transfusion of at least 1 unit of packed red blood cells within a 24-h period. The secondary outcome was defined the length of stay (LOS) during index hospitalization.

### Statistical analysis

Baseline characteristics are presented as means (±Standard deviation) or medians (±interquartile range). Comparisons between two groups characteristics were performed using the Student’s t-test or Chi square for continuous and categorical variables respectively; ANOVA was used for non-parametric variables. Length of stay between the two groups has been plotted as median (± interquartile range) in a Whisker plot. Odds ratios have been computed for comparison of outcomes. Two separate analyses have been performed to accommodate for the different times points that the population was studied (index admission [immediate postoperative] vs. 3-months follow up [early postoperative]). Adjusted odds ratios have been also computed to account for baseline characteristics that could affect outcomes (age, sex, race, type of LVAD, INTERMACS classification, type of cardiomyopathy, coronary artery disease, prior stroke, chronic kidney disease, atrial fibrillation, hypertension, diabetes mellitus, obesity and obstructive sleep apnea). A *p*-value ≤0.05 was considered statistically significant. All statistical analyses were performed using the Stata (StataCorp; College Station; TX).

## Results

### Baseline characteristics

**Fig. 1:**
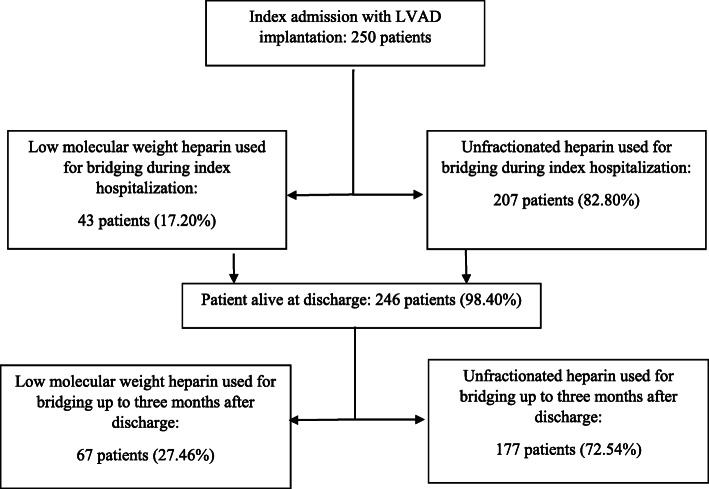
Study flow diagram

A total of 250 patients that underwent LVAD implantation with HVAD (*n* = 102), HeartMate II (*n* = 69) or HeartMate III (*n* = 79) were included in the analysis during their index hospitalization (Fig. 1). Patients in the UFH group were older in age (56.1 ± 11.96 years vs 51.9 ± 15.3 years, *p* = 0.02), more likely to be male (82% vs 67%, *p* = 0.03), Caucasian (80.7% vs 65.1%, p = 0.03) and have paroxysmal atrial fibrillation (41% vs 16%, *p* = 0.005). Patients in the enoxaparin group were more likely to have chronic kidney disease than those in the UFH arm (41.9% vs 15.9%, *p* < 0.01). Baseline characteristics are detailed in Table 1.

**Table 1:** Baseline Characteristics during Index hospitalization and follow-up

	LMWH Index ***N*** = 43	UFH Index ***N*** = 207	P	LMWH follow-up ***N*** = 67	UFH follow-up ***N*** = 177	P
**Demographics**						
Age (SD)	51.9 (15.3)	56.1 (12.0)	0.02	52.7 (13.8)	56.6 (12.0)	0.02
Males (%)	29 (67.4)	170 (82.1)	0.03	58 (74.4)	141 (82)	0.17
Race (%)			0.03			0.001
Caucasian	28 (65.1)	167 (80.7)		50 (64.1)	145 (84.3)	
African American	13 (30.2)	39 (18.8)		26 (33.3)	26 (15.1)	
Others	2 (4.7)	1 (0.5)		2 (2.6)	1 (0.6)	
**Type of LVAD (%)**			0.13			0.07
HeartWare	18 (41.9)	84 (40.6)		36 (46.2)	66 (38.4)	
HeartMate II	17 (39.5)	52 (25.1)		25 (32.1)	44 (25.6)	
HeartMate III	8 (18.6)	71 (34.3)		17 (21.8)	62 (36.1)	
**INTERMACS classification (%)**			0.55			0.18
INTERMACS 1	7 (16.3)	33 (15.9)		11 (14.)	29 (16.9)	
INTERMACS 2	13 (30.2)	40 (19.3)		24 (30.8)	29 (16.9)	
INTERMACS 3	17 (39.5)	110 (53.1)		37 (47.4)	90 (52.3)	
INTERMACS 4–7	6 (14.0)	24 (12.0)		6 (7.7)	24 (14.0)	
**Comorbidities (%)**						
Cardiomyopathy			0.32			0.38
Ischemic	17 (39.5)	99 (47.8)		33 (42.3)	83 (48.3)	
Non-ischemic	26 (60.5)	108 (52.2)		45 (57.7)	89 (51.7)	
CAD	20 (46.5)	75 (36.2)	0.21	34 (44.0)	61 (35.5)	0.22
Prior stroke	7 (16.3)	30 (15.0)	0.76	14 (18.0)	23 (13.34)	0.35
CKD	18 (42.0)	33 (16.0)	< 0.01	30 (38.5)	21 (12.2)	< 0.01
Atrial fibrillation			0.005			< 0.01
Paroxysmal	7 (16.3)	85 (41.1)		16 (20.5)	76 (44.2)	
Permanent	3 (6.9)	5 (6.9)		6 (7.7)	2 (1.2)	
Hypertension	29 (67.4)	128 (61.8)	0.49	55 (70.5)	102 (59.3)	0.09
Diabetes mellitus	18 (41.9)	102 (49.3)	0.38	32 (41.0)	88 (51.2)	0.14
Obesity	11 (25.6)	80 (38.8)	0.10	27 (34.6)	64 (37.4)	0.67
OSA	9 (20.9)	44 (21.4)	0.95	23 (30.0)	30 (17.5)	0.03

*CAD* Coronary Artery Disease, *CKD* Chronic Kidney Disease, *LVAD* Left Ventricular Assist Device, *OSA* Obstructive Sleep Apnea

Among 246 individuals followed for 3 months post LVAD implantation, patients in the LMWH arm were significantly younger (52.7 years vs. 56.6 years, *p* = 0.02), and more likely to have obstructive sleep apnea (29.5% vs 17.5%, *p* = 0.03), permanent atrial fibrillation (7.7% vs 1.2%, *p* < 0.001) and chronic kidney disease (38.5% vs 12.2%, *p* < 0.01). Patients in the UFH group were more likely to be Caucasian (84.30% vs 64.10%, *p* = 0.001), have an HM III implanted (36% vs 21.7%, *p* = 0.07) and have paroxysmal atrial fibrillation (44.2% vs 20.5%, *p* < 0.01).

### Outcomes

#### Outcomes during index hospitalization

There was a total of 5 (11.6%) bleeding events in the LMWH group vs 24 (11.6%) in the UFH group. With regards to CVA there was 1 (2.3%) event in the LMWH group as compared to 11 (5.3%) events in the heparin group (Central Illustration). The calculated OR for bleeding events was 1.01 (CI = 0.36–2.79, *P* = 0.99) and for CVAs was 0.42 (CI =0.05–3.38, *P* = 0.42). When adjusted for age, sex, race, type of LVAD, INTERMACS classification and comorbidities, the ORs remained 0.91 (CI = 0.27–3.04, *P* = 0.88) and 0.67 (CI = 0.07–6.39, *P* = 0.73) for bleeding and CVAs respectively (Table 2). No fatal thromboembolic or hemorrhagic events occurred.

**Table 2:** Bleeding events and Cerebrovascular accidents

	LMWH	UFH
**Index admission**	***N*** **= 43**	***N*** **= 207**
Bleeding (%)	5 (11.6)	24 (11.6)
CVA (%)	1 (2.3)	11 (5.3)
**Follow-up**	***N*** **= 67**	***N*** **= 181**
Bleeding (%)	7 (10.5)	27 (14.9)
CVA (%)	1 (1.5)	0 (0)

*CVA* cerebrovascular Accident, *LMWH* Low Molecular Weight Heparin, *UFH* unfractionated heparin

#### Outcomes during 3 month-follow up period

A total of 7 (10.5%) bleeding events occurred in the LMWH group compared to 27 (15%) in the UFH group (Table 2). Accordingly, there were 1 (1.5%) and 0 (0%) CVAs in the LMWH and UFH groups respectively (Central Illustration). The unadjusted OR for bleeding events was 0.67 (0.28–1.61; *P* = 0.37) and did not reach statistical significance after adjustment (OR = 0.85; 0.31–2.34; *p* = 0.76). No OR for CVAs was computed due to low event rates (Table 3). Similarly, there were no fatal events during the 3-month follow-up period.

**Table 3:** Cumulative, index admission and 3-month follow-up odds ratios

Outcomes	Unadjusted OR (CI)	P	Adjusted OR (CI)^**a**^	P
**Cumulative**				
Bleeding events	1.12 (0.6–2.2)	0.73	1.2 (0.5–2.8)	0.62
CVA	0.39 (0.1–1.8)	0.22	0.42 (0.1–2.6)	0.35
**Index**				
Bleeding events	1.01 (0.4–2.8)	0.99	0.91 (0.3–3.0)	0.88
CVA	0.42 (0.6–3.4)	0.42	0.67 (0.1–6.4)	0.73
**Follow-up**				
Bleeding events	0.67 (0.3–1.6)	0.37	0.85 (0.3–2.3)	0.76
CVA	n/a	n/a	n/a	n/a

^a^Adjusted for age, sex, race, type of LVAD, INTERMACS classification, type of cardiomyopathy, coronary artery disease prior stroke, chronic kidney disease, atrial fibrillation, hypertension, diabetes mellitus, obesity and obstructive sleep apnea

*CI* Confidence Interval, *CVA* Cerebrovascular Accident, *OR* Odds Ratio

#### Cumulative odds ratios for total study period

The cumulative odds ratio for bleeding events in patients receiving LMWH for the total study period including Index hospitalization and follow-up 3 months period was 1.12 (CI = 0.57–2.24, *P* = 0.73). When adjusted for age, sex, race, type of LVAD, INTERMACS classification and comorbidities, the OR remained 1.2 (CI = 0.54–2.84, *P* = 0.62). The OR for CVA amongst LMWH patients during the study period was 0.39 (CI = 0.08–1.78, *P* = 0.22) and adjusted OR of 0.42 (CI = 0.07–2.59, *P* = 0.35).

#### Length of in-hospital stay

**Fig. 2:**
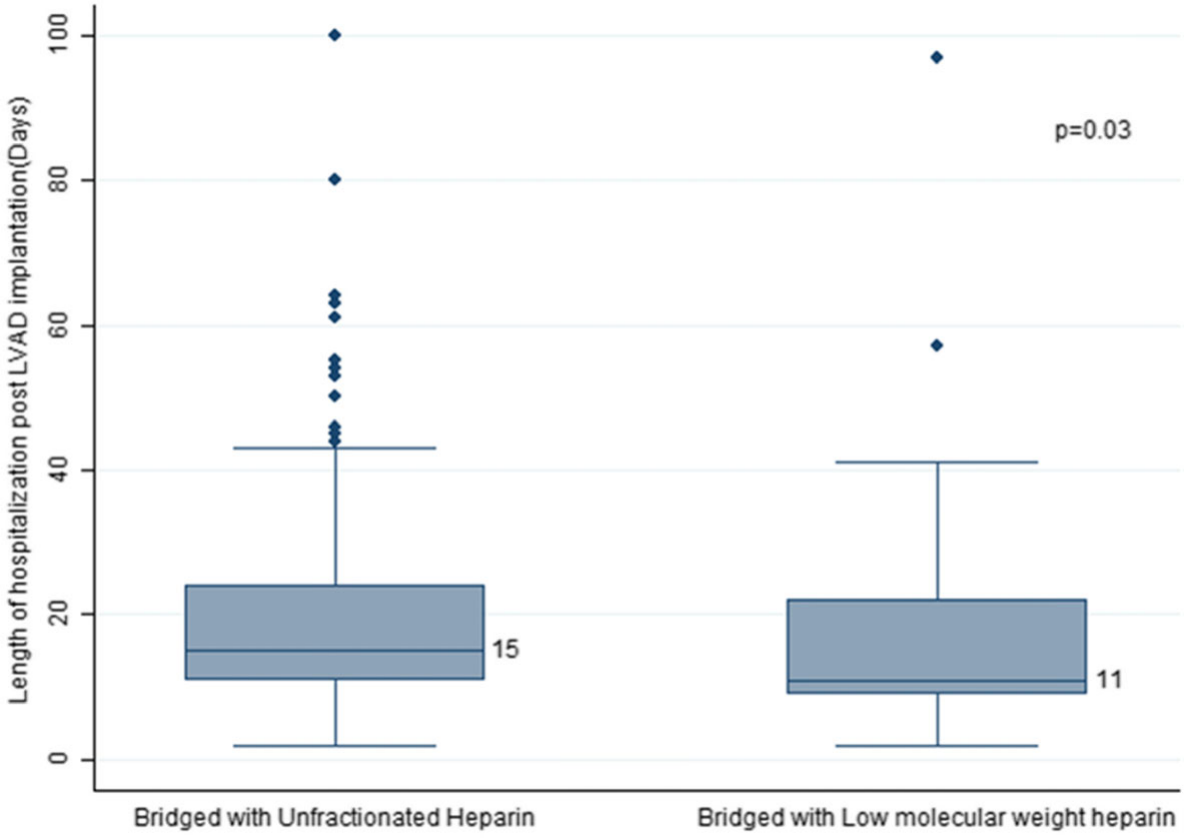
Median length of stay during index hospitalization after LVAD implantation

Analysis of patient’s in-hospital stays showed that the median length of stay during index hospitalization in the LMWH group was 11 days (IQR 9–22) compared to 15 days (IQR: 11–24; *p* = 0.03) in the UFH group resulting in a statistically significant 4-day shorter in-hospital stay postoperatively. A Box and Whisker plot illustrating median value, interquartile range and outliers is depicted in Fig. 2.

## Discussion

This is the first study comparing the safety and efficacy of enoxaparin with unfractionated heparin in the immediate and early post-operative period after LVAD implantation. The main finding of our analysis is that LMWH is purported as both safe with regards to bleeding and efficacious in preventing CVAs when compared to intravenous anticoagulation with UFH as a bridge to therapeutic INR. It is worth mentioning that despite the fact that differences did not reach statistical significance likely due to low sample size, a trend towards lower bleeding events was observed in the LMWH group (10.5% vs 15%) compared to their UFH counterparts in the early postprocedural period.

More importantly, we found that comparison of in-hospital stays between the two groups has shown a statistically significant shorter hospital stay of 4 days in the LMWH group (11 days [IQR 9–22] vs 15 days [IQR: 11–24]; *p* = 0.03) when compared to UFH group. This comes as an expected result of the LMWH use profile and medication pharmacologic properties. In addition, LOS has direct implications in reducing post-procedural complications from prolonged hospitalization (infections, further deconditioning, etc.) as well in reducing hospitalization costs. In a recent analysis from the INTERMACS database, 13,705 patients were stratified in 3 groups based on their in-hospital length of stay (> 30 days, 30–60 days, > 60 days) post LVAD implantation [18]. The study found a gradual increase in mortality (20% vs 26% vs 58%) with prolonged hospitalization highlighting the benefit of short LOS in this surgical population.

LMWH is often used as a bridge to therapeutic INR among patients requiring anti-coagulation for various indications [12]. The main advantage of this strategy is that once provided with required education and training, patients can self-administer this medication and do not have to remain hospitalized awaiting therapeutic INR. Furthermore, the onset of action of LMWH is rapid, with excellent and consistent bioavailability ensuring full and predictable therapeutic anti-coagulation soon after administration [19, 20]. These pharmacologic properties could partially explain the numeric differences in bleeding events in the two groups given the inconsistent bioavailability and variable degrees of anticoagulation with heparin. A major disadvantage of an enoxaparin strategy is that although its effects are partially reversed by protamine sulfate, no complete reversal agent currently exists leading to potential catastrophic outcomes. This is especially relevant in the immediate post LVAD implantation period when the risk of surgical and wound bleeding is not trivial. Hence, the bulk of existing evidence of LMWH as a bridging strategy refers to the outpatient setting among patients with more than 3 months passed from LVAD implantation.

Despite the clear indication for uninterrupted and carefully monitored therapeutic anti-coagulation for prevention of LVAD thrombus, a recent INTERMACS report found that up to 25% of LVAD patients had an INR between 1.4–1.6 over their 4 years study period [3]. Najjar et al. also found that patients who had been diagnosed with LVAD thrombus were only at a therapeutic window 40.5% of the time [21]. Owing to the significant amount of time LVAD patients find themselves with subtherapeutic levels of anticoagulation, there has been a growing interest in the utilization of low molecular weight heparin (LMWH) in the outpatient setting as a bridge to therapeutic INR. Schisler et al. at demonstrated successful utilization of enoxaparin as a bridge in 110 subtherapeutic patients over an average duration of 3 days without an increase in bleeding or thrombotic events [21, 22]. Borden et al. also showed similar results in their patient population [23]. However, in both these studies patients being bridged to therapeutic INR with enoxaparin were at least 3 months out from their LVAD implantation.

The notion of using LMWH early in the postoperative period after LVAD implantation is not completely novel but large-scale comparative studies among different regimens are lacking. Sandner et al. evaluated the feasibility of LMWH use in the post-operative period [24]. This was a single arm study of 78 patients that underwent LVAD placement and received AC with either enoxaparin or dalteparin. The investigators observed only 3 CVAs and 5 bleeding events in the post-operative period on enoxaparin without any fatal events. They subsequently concluded that LMWH is a feasible option post LVAD as a bridging strategy; however, the absence of a comparative arm makes these findings less reliable. In a similar but smaller study of 8 patients by the same investigator published in 2008, nadroparin was used in the post-operative period after LVAD implantation [25]. While there were 2 ischemic CVAs and 2 suspected pump thromboses, no bleeding was reported. Furthermore, long-term anticoagulation with LMWH has been also found to be feasible in patients with implantable LVAD as an alternative to oral AC as bridge to transplantation approach [26]. In contrast to all these previous reports, a recent retrospective study of 118 patients by Bhatia et al. found that a bridging strategy with enoxaparin as compared to no bridging in patients with subtherapeutic INR was associated with a four-fold increase in major bleeding events with bridged vs non-bridged period within the enoxaparin group. However, no differences were identified between the enoxaparin and the non-bridge groups [27]. Current guidelines (presented earlier) recommend the use of parenteral anticoagulation as a bridge strategy in both post-op and long-term period. Our study provides the most definite evidence of enoxaparin safety and efficacy in this specific patient population.

The use of LMWH in the immediate postoperative period of open-heart surgery has been previously shown. Feasibility of LMWH in the setting for mechanical heart valve replacement has been evaluated in two reports by Montalescot and Meurin et al. published in 2000 and 2006 respectively [16, 17]. Both studies combined amassed approximately 900 patients including patients with traditional thromboembolic risk factors such as atrial fibrillation and hypertension. Bridging AC strategy with enoxaparin was found to be feasible with only few major bleeding events and no perceived increase in thromboembolic events. Importantly no fatal events occurred during a 90-day follow up period. More recently, Kindo et al. performed a prospective, single arm study of 1063 consecutive patients undergoing mechanical heart valve replacement with postoperative LMWH AC treatment [15]. Patients were followed for 6 weeks post-surgery. LMWH was initiated in the first day after surgery and continued until therapeutic INR was achieved. Approximately 1% of patients had a CVA whereas 4.1% experienced major bleeding. No fatal events were noted in follow-up period.

There are some important limitations of our study. Firstly, our study is a retrospective analysis of consecutive patients cared for in our medical centers. As such, it is not possible to completely rule out unidentified confounding factors that would account for the findings of our study. Furthermore, our findings may be highly subject to selection bias of patients deemed suitable for the LMWH arm, especially when considering 3 different LVADs with differing risk profiles were utilized in this patient population. However, our study is in accordance with previous ones (both in LVAD and mechanical valve populations) showing the safety of LMWH in the postoperative period. Additionally, the generalizability of our findings is limited by the fact that our study is limited to 2 quaternary level medical centers that may have varying intraoperative, perioperative and post-operative surgical and medical management strategies from not only each other, but the LVAD community at large. Next, given the retrospective nature of our study, it is difficult to establish causality for the reduction of length of stay in the LMWH arm albeit it is reasonable to extrapolate causality in this setting. Finally, the relatively small sample size in our study warrants larger randomized controlled studies to confirm our findings.

## Conclusions

In these double center, retrospective study of 250 consecutive LVAD patients we found that LMWH is an equally effective, and possibly safer, strategy as compared to UFH in bridging post LVAD patients to therapeutic INRs in the immediate and early post-operative period. Furthermore, it significantly reduces the length of stay during index hospitalization. Further large-scale studies are needed to establish our findings.

## Data Availability

Data used for this study are available upon request.

## Abbreviations

AC: Anticoagulation
CVA: Cerebrovascular Accident
INR: International Normalized Ratio
IV: Intravenous
INTERMACS: Interagency Registry for Mechanically Assisted Circulatory Support
LMWH: Low Molecular Weight Heparin
LVAD: Left Ventricular Assist Device
UFH: Unfractionated Heparin
