# Correction to: The use of enoxaparin as bridge to therapeutic INR after LVAD implantation

**DOI:** 10.1186/s13019-021-01451-9

**Published:** 2021-04-14

**Authors:** Zubair Shah, Ioannis Mastoris, Prakash Acharya, Aniket S. Rali, Moghni Mohammed, Farhad Sami, Sagar Ranka, Savahanna Wagner, Giorgio Zanotti, Christopher T. Salerno, Nicholas A. Haglund, Andrew J. Sauer, Ashwin K. Ravichandran, Travis Abicht

**Affiliations:** 1 Department of Cardiovascular Medicine, University of Kansas Health System, University of Kansas School of Medicine, Kansas City, Kansas, USA; 2 Cardiovascular Service Line, Ascension, St. Vincent Hospital, Indianapolis, Indiana, USA; 3 Department of Cardiovascular and Thoracic Surgery, University of Kansas Health System, University of Kansas School of Medicine, Kansas City, Kansas, USA

**Correction to: J Cardiothorac Surg 15, 329 (2020)**

**https://doi.org/10.1186/s13019-020-01373-y**

The original article [1] incorrectly inverted co-author, Farhad Sami’s name. This has since been corrected.
